# Crime and Punishment
*"Crime, its Amount, Causes, and Remedies." By Frederick Hill, late Inspector of Prisons. John Murray. 1853.


**Published:** 1853-04-01

**Authors:** 


### CRIME AND PUNISHMENT.*

We received Mr. Hill's valuable and elaborate essay at too late a period to make a careful analysis of its contents in time for publication in the present number of our journal. We must satisfy ourselves by making a few extracts from the work, reserving for our succeeding number some critical remarks.

### DECREASE IN THE AMOUNT OF CRIME.

"I am happy to be able to state, as the result of many years inquiry and observation, that my belief is, that even under present circumstances, the quantity of crime in this country is steadily decreasing and taking a milder and milder form; that it is less than at any previous period of our history, even without reference to the increase of wealth and population; but that bearing these in mind, and estimating the extent of crime by the average amount of privation, fear, and suffering which it causes to each member of society, the decrease is great indeed."

"As examples of the small numbers of habitual offenders, it will be seen by a reference to my Second Report that at that time (1836) there were only nine resident thieves at Kinghorn, in Fife, where the population was 1500; and that there were not more than about twelve in the whole of East Lothian, where the population was about 36,000."

"This small number of offenders is not inconsistent with a large number of offences; for in the same way as a great many hats are made by a single hatter, and a great many shoes by a single cobbler, so a great many thefts are committed by a single thief. Thus, in my Fourth Report a case is mentioned, which will be more particularly noticed hereafter, where it is estimated that as many as twenty thousand separate offences were probably committed from first to last by only fifteen persons. Many similar instances appear in my other reports, and in the reports of Mr. Clay, the zealous, benevolent, and able chaplain of the prison at Preston."

### PRESENT MODE OF CHECKING CRIME.

"It is still too much directed to detection and too little to prevention, thus reserving high rewards till great offences have been committed, instead of making all reward simply dependent on the small amount of crime.

"So long as the police receive large premiums for the apprehension of great criminals it is evidently their interest (although many of them are, no doubt, too honourable to be so swayed) that great criminals should exist; their motive for the extinction of such offenders being scarcely greater than that of a poacher for the extirpation of hares and pheasants. And great as is this objection to the practice of offering such rewards, it is not the greatest; for the terrible cases of "blood-money" that have sometimes come to light, show that official villains have been found, under the stimulus of these rewards, to get up evidence against persons who were wholly innocent.

"One great advantage of a register of crime, such as I have described, would be the means thereby afforded of regulating the payments to the police on sound principles.

"With a good system for paying the police, and with other good general arrangements, powerful motives would be created for striking at the

* "Crime, its Amount, Causes, and Remedies." By Frederick Hill, late Inspector of Prisons. John Murray. 1853.

main roots of crime by seizing on the receivers of stolen goods—the burglar capitalists—instead of directing so much attention to the apprehension of single offenders.

"The following evidence given to me by an intelligent prisoner, an inmate of the Edinburgh Bridewell, before the reform of that prison, appears in my report for 1838-9:—

"Has had a great deal of conversation with thieves of all ages, who have come to the Bridewell, and the following is the result of the information he has received:—Stealing is generally done systematically, and as a matter of regular business. Most of the thieves know each other, and know the circumstances of the various robberies that are committed. They associate much together. The chief body of the thieves in Edinburgh live in the wynds and closes out of High-street. With vigour and talent on the part of the police, and with proper prisons to receive the offenders, the whole system might be knocked on the head. There are four or five houses which the thieves have frequently mentioned where they can get food, drink, and lodging on credit, it being well known how they are to procure the means of paying. At these houses also they meet girls of ill-fame. All these houses are situated either in the High-street, or in wynds leading from it. They serve as nests and centres of crime. Many of the young thieves have also mentioned public-houses where they are allowed to drink to any extent, the characters of whose landlords declarant had always supposed to be respectable. There is no difficulty of disposing of stolen goods. Women frequently act as agents for young thieves. There is one notorious person named ——, living on the south side of the town, who pretends to be a travelling jeweller, but who is in fact a receiver of stolen goods. Every one going to dispose of stolen articles, gives a peculiar knock at his door. A little boy, who lately slept with declarant, told him that he had sold a watch to this man for 9*s*., which he had stolen from his brother-in-law, and which he believed to be worth 6*l*."

"An important advantage arising from vigorous measures to reduce the number of criminals at large, would be, that the situation of every remaining criminal would become more difficult.

"Some persons, indeed, have fears that to withdraw a number of criminals is only to create a void which will call new criminals into existence. But this appears to me to be a great mistake; founded probably on a false analogy between crime and food, or some other thing for which there is a natural demand, the pressure of which would increase with any diminution in the number of producers. Such, however, is not the case with crime, for crime is opposed to the interests and feelings of society; and the community at large would be glad to drive it out of existence; and instead of the expansive power of crime increasing with every contraction, I believe the reverse to be the case, and that the withdrawal of every criminal, inasmuch as it tends to break up organization for dishonest purposes, and to bring each remaining offender more prominently before the public eye, weakens the position of criminals generally, and facilitates the complete suppression of crime. It is this want of a crowd of offenders to afford the means of a division of labour and mutual concealment that, in my opinion, does much to account for the almost total absence of crime in many small places."

### SUGGESTIONS FOR THE PREVENTION OF CRIME.

"Some of the most objectionable taxes, in their tendency to create crime, are those which afford temptations to steal money. As regards cheques, indeed, provided they are to be paid through a bank, a security has of late years been devised; but no such safeguard has yet been extended to banknotes, though this might readily be done if there were no stamp duty to prevent ordinary bank-notes being made payable, like bank post-bills, a certain number of days after sight.

"If this change were made, and if the law allowed the same free use of paper money in the rest of the kingdom as it does in Scotland, there would seldom be much temptation, so far as money is concerned, to turn highwayman, footpad, or burglar; for the money, when obtained, would in the main be of no value, as its payment could instantly be stopped. The common offence, also, of stealing from letters would gradually disappear; and the public would be afforded the great convenience, which they have not at present, of being able to transmit money by letter without danger."

### SOCIAL POSITION OF WOMEN IN RELATION TO CRIME.

"While we may congratulate ourselves on the great erasures from the class of unjust laws, tending to create habits of crime, we must still turn with shame and regret to the state of deprivation, amounting, not unfrequently, to positive servitude, in which we find so many of our humble countrywomen. The married woman, in the lower classes, is in effect so indissolubly bound to the man whom she has once received as her husband, that whatever may be his offences against conjugal fidelity, sobriety, honesty, kindness, or duty of any kind, so long as they do not place him immediately within the grasp of the law, she has no protection either for herself or her children against any wrongs he may think proper to inflict. How many poor wives are there who would most cheerfully and effectually maintain themselves and their children, in other words, do their husband's whole duty, if they could but be guaranteed against his violence and dishonesty! Application for legal redress avails little or nothing. To obtain divorce, or even separation, involves an expense beyond the total earnings of years—perhaps of a whole life—and complaint to a magistrate, the only process open, obtains at best but a temporary relief, followed, in all probability, by more malicious, if less open ill-treatment.

"How often does it occur, that, after the husband has absconded for years, during which the deserted wife, by painful toil and rigid self-denial, has kept her family in decency, and got together some little store, he has returned only to destroy her comforts, sell up her little furniture, and strip her even of those very implements by which alone she can earn her own and her children's bread!

"The profligate, returning from an adulterous life—the brutal soldier, discharged for misconduct—the very convict, released from transportation, comes back in full authority to despoil and oppress the wife whom he ought to have cherished, and the children whom he should have reared.

"During the time of my inspection in Scotland, many, I believe the majority, of the murders that were committed were those of wives and husbands; most of which would probably have been prevented could the suffering party have obtained a separation.

"The State of New York, which lately set so good an example to this country in the junction, as respects procedure, of what, it may be hoped, will one day be always found united in another sense besides that implied by the 'fusion' of law and equity, has now made an advance worthy of general imitation, towards rendering legal justice to women, by decreeing that the property of a married woman in New York shall, without special covenant, be at her own disposal, instead of being handed over to her husband.

"A striking instance of the evils resulting from the want of the means of obtaining a divorce under proper circumstances, and of the injustice caused by the uncertainty and conflicting character of some of our laws, is afforded by the case of Lolly, which, though tolerably familiar in its early stages to the legal profession, is not, I believe, generally known in its sequel even to lawyers. A person of this name having married in England, afterwards procured a divorce in Scotland; such a proceeding, when there is good ground for it, being there within the reach of any one of moderate property, though beyond that of the poor. After a time Mr. Lolly married again; and, in the words of Lord Brougham, when speaking as Lord Chancellor, in so doing he acted '*boná fide*, and in the confident belief, founded on the authority of the Scotch lawyers, that the Scotch divorce had effectually dissolved his prior English marriage.' Nevertheless he was brought to trial at Lancaster for bigamy, convicted, and *transported*.

"After the expiration of his period of punishment, Mr. Lolly, while still in Australia, by industry and perseverance, acquired a considerable property for a person in his rank of life, and at length returned to England; but hearing, on his arrival, that his second wife had, in his absence, married again, he was so bitterly disappointed and distressed, that he destroyed himself. After his death, notwithstanding her subsequent marriage, this second wife claimed in the Ecclesiastical Court the right of administration, and in effect that of succession to his property; and after a full hearing of the cause, judgment was given in her favour; thus reversing, as far as it was competent to this Court, the verdict of the Criminal Court, and awarding a degree of wealth to one partner of an act, though the other partner of the same act had been disgraced, banished from his native country, and condemned to associate for years with felons."

We have so frequently urged the necessity for a new law of partnership, that we need only quote this further testimony:—

### LAW OF PARTNERSHIP.

"In the category of laws tending to produce crime, although its evils may not be equally manifest, I should put the present law of partnership; for this law, by rendering partnerships dangerous, tends, perhaps, more than anything else, to separate the employers and the employed; to prevent the sympathy and union of interests which might otherwise exist between them; to check, among the workmen, the growth of the feeling of self-respect (so moral and so truly conservative in its tendency) which results from the sense of proprietorship; and to give rise to that system of tyranny to which I have already referred, and wherein bodies of workmen attempt, by violence and terror, to deter their fellow-operatives from obtaining a living, except on such terms as they, the dictators, may choose to prescribe.

"At present, as is well known, an employer dares not admit his workmen into any degree of partnership, because thereby his whole property might be jeopardized; but if, as the great principle of non-interference to which this country owes so much of its energy and superiority would dictate, people were allowed to form partnerships on what terms they pleased, partnerships with limited liabilities would probably soon become general; and into these, under judicious regulations, any number of people might safely be received.

"The only cases in which it seems proper for the law to interfere are where persons attempt to mislead the public as to the real terms of their partnership, and thereby to obtain money or credit on false pretences; or where they evade the agreements they have entered into.

"On such an arrangement as that contemptated, a workman might put 10*l.*, 20*l.*, 50*l.*, or 100*l.* into the concern in which he was employed, and to that extent become a partner; receiving, in addition to his wages as a workman, a proportionate share of the general profits. And no doubt the chief proprietor would soon find his account in this; for, besides being relieved from the anxiety attendant on having his whole property at stake, he would partake largely of the benefit resulting from an increased energy in production, a greater spirit of economy, and a freedom from the interruptions and losses consequent on strikes. The late unhappy and formidable differences between the mechanic engineers and their employers, which brought to light errors on both sides, though much good also, and an evident desire in each party not to exceed its rights, would probably never have arisen had there existed a good law of partnership, and of sufficient age to have had time to work its way into practice, and into a right understanding with employers and employed."

### LUNATIC ASYLUMS.

"No one thinks of sending a madman to a lunatic asylum for a certain number of days, weeks, or months. We content ourselves with carefully ascertaining that he is unfit to be at large, and that those in whose hands we are about to place him act under due inspection, and have the knowledge and skill which afford the best hope for his cure; that they will be kind to him, and inflict no more pain than is necessary for his secure custody and the removal of his malady; and we leave it for them to determine when he can safely be liberated.

"It is true that, great as have been the improvements of late years in lunatic asylums generally, and admirably as some of them are now conducted, there are still many and great abuses. But, however much these abuses may be condemned, no one for a moment suggests, as a consequence of their existence, that madmen should henceforth be subjected only to specified periods of confinement. Instead of this the public demands, and rightly demands, that a more efficient system of inspection should be established, and that the governors and managers of lunatic asylums should be held to a stricter responsibility.

"Perhaps it may ultimately be found, by cautious experiment, that a somewhat similar process may be safe and expedient in the treatment of criminals; and that while it is still left to the courts of justice to determine on the guilt or innocence of the accused, and on the necessity of their withdrawal from society, it may be assigned to those intrusted more or less directly with the reformatory treatment to determine the time of release; subject, however, to a most competent, well-appointed, careful, and responsible supervision and control, such as ought to be invariably exercised in the case of madhouses; and subject to the proviso, that no amount of subsequent good conduct should be considered sufficient to warrant the liberation of a person who had once been guilty of deliberate murder."

### PRISON DISCIPLINE.

"But let the visitor reflect that, first, as respects the honest workman, the prisoner has entirely lost his freedom, and ceased to be his own master; that he is not only entirely cut off from family and friends, but that, generally, he is deprived of companionship altogether; that he must neither whistle, sing, nor shout; that, day after day, and month after month, except at the intervals of exercise, he is confined within the four walls of his little cell, Sundays and holidays affording no relief, the very changes of the season almost unknown to him, for all, at least, that he can partake of their charms,—let him think of this, and he will probably be of opinion that, though the prisoners were fed on turtle, instead of barley broth, and slept on down, instead of straw, there would still be few applicants among the honest working class for permission to occupy their places.

"And let the visitor, further, make himself acquainted with the habits of criminals, and with their ideas of comfort and luxury, and he will probably come to the conclusion that their distress must indeed be severe, and such as to make their being at large dangerous to all around them, before such persons would voluntarily enter a prison.

"For what, owing generally to wretched training, are the habits of criminals? Idleness, late rising, and indulgence in drinking, smoking, and gambling. And what regard is paid to these habits, however strong they may be, on entering a Scottish prison? Not the slightest. However great a sluggard, he must rise, the very morning after his admission, even in the middle of winter, when the clock strikes six. Then, although he would probably prefer remaining in his dirt to the trouble of making himself clean, he must immediately wash himself, and that thoroughly. So soon as that is done, he must, if he has been tried, begin a task of labour, with the prospect of losing his dinner if he be sullen and refuse to complete it. Should he ask for a companion he will be at once refused. Between times he may wish to comfort himself with a pipe, or at least with a pinch of snuff; but no, the rules inexorably and most properly forbid all luxuries, especially such as foster habits of expense. At dinner, he may ask for at least a little beer; but he is again refused, and he finds that, however much against his will, he has suddenly become a member of a total abstinence society. As for opportunities of gambling, he has neither anything to stake nor any person with whom to play.

"When it is considered how painful an effort is generally necessary to break through a single bad habit, it may be judged how much a person, under such circumstances, must suffer; and it will be seen that that which is pleasing to the eye of the visitor, and excellent in itself, is often obtained with much, though necessary, pain; and the delusion will be dispelled that the prisons have ceased to be places of punishment."

---

